# Efficiency of Neat and Quaternized-Cellulose Nanofibril Fillers in Chitosan Membranes for Direct Ethanol Fuel Cells

**DOI:** 10.3390/polym15051146

**Published:** 2023-02-24

**Authors:** Maša Hren, Damjan Makuc, Janez Plavec, Michaela Roschger, Viktor Hacker, Boštjan Genorio, Mojca Božič, Selestina Gorgieva

**Affiliations:** 1 Faculty of Mechanical Engineering, University of Maribor, Smetanova 17, 2000 Maribor, Slovenia; 2 Slovenian NMR Centre, National Institute of Chemistry, Hajdrihova 19, 1000 Ljubljana, Slovenia; 3 EN-FIST Centre of Excellence, Trg Osvobodilne fronte 13, 1000 Ljubljana, Slovenia; 4 Faculty of Chemistry and Chemical Technology, University of Ljubljana, Večna pot 113, 1000 Ljubljana, Slovenia; 5 Institute of Chemical Engineering and Environmental Technology, Graz University of Technology, Inffeldgasse 25/C, 8010 Graz, Austria; 6 Dravske Elektrarne Maribor d.o.o., Obrežna ulica 170, 2000 Maribor, Slovenia

**Keywords:** chitosan, cellulose nanofibrils, anion exchange membrane, direct alkaline alcohol fuel cell

## Abstract

In this work, fully polysaccharide based membranes were presented as self-standing, solid polyelectrolytes for application in anion exchange membrane fuel cells (AEMFCs). For this purpose, cellulose nanofibrils (CNFs) were modified successfully with an organosilane reagent, resulting in quaternized CNFs (CNF (D)), as shown by Fourier Transform Infrared Spectroscopy (FTIR), Carbon-13 (C13) nuclear magnetic resonance (^13^C NMR), Thermogravimetric Analysis (TGA)/Differential Scanning Calorimetry (DSC), and ζ-potential measurements. Both the neat (CNF) and CNF(D) particles were incorporated in situ into the chitosan (CS) membrane during the solvent casting process, resulting in composite membranes that were studied extensively for morphology, potassium hydroxide (KOH) uptake and swelling ratio, ethanol (EtOH) permeability, mechanical properties, ionic conductivity, and cell performance. The results showed higher Young’s modulus (119%), tensile strength (91%), ion exchange capacity (177%), and ionic conductivity (33%) of the CS-based membranes compared to the commercial Fumatech membrane. The addition of CNF filler improved the thermal stability of the CS membranes and reduced the overall mass loss. The CNF (D) filler provided the lowest (4.23 × 10^−5^ cm^2^ s^−1^) EtOH permeability of the respective membrane, which is in the same range as that of the commercial membrane (3.47 × 10^−5^ cm^2^s^−1^). The most significant improvement (~78%) in power density at 80 °C was observed for the CS membrane with neat CNF compared to the commercial Fumatech membrane (62.4 mW cm^−2^ vs. 35.1 mW cm^−2^). Fuel cell tests showed that all CS-based anion exchange membranes (AEMs) exhibited higher maximum power densities than the commercial AEMs at 25 °C and 60 °C with humidified or non-humidified oxygen, demonstrating their potential for low-temperature direct ethanol fuel cell (DEFC) applications.

## 1. Introduction

Fuel cells are an advantageous, clean, and efficient technology for power generation. The alkaline medium of anion exchange membrane fuel cells (AEMFCs) offers advantages in cost, stability, and durability, due primarily to the use of non-precious metal catalysts, as well as ease of handling, storage, and transportation of liquid fuels, which can facilitate mature commercialization compared to gaseous hydrogen. One of the device-level challenges in alkaline fuel cells is membrane selection. Membranes in the form of solid polymer electrolytes are a crucial segment of AEMFCs. They consist of a hydrophobic polymer backbone with positive hydrophilic groups that function according to the Donnan exclusion principle [1] and serve as a barrier between the anode and cathode while conducting ions and suppressing the passage of electrons [2,3]. The first anion exchange membrane (AEM), developed by the Tokuyama Corporation (Japan) consists of polychloroprene as a polymer backbone crosslinked with divinylbenzene and containing quaternary ammonium functional groups such as tri-ethylamine [4]. Since then, various types of AEMs have been developed, such as poly(vinyl alcohol) (PVA) [5], poly(ether ketone) [6], poly(ether ether ketone) (PEEK) [7], poly(ether sulfone) (PES) [8], poly(vinylidene fluoride) (PVDF) [9], and polyetherimide (PEI) [10]. As can be seen, the field of AEMs is covered commercially entirely by the fully synthetic membrane types. In contrast, biopolymer-based materials intended for such use have yet to be developed to meet the stringent requirements for commercial use.

Chitosan (CS) has been introduced into fuel cell research as an environmentally friendly, abundant biopolymer. This amino polysaccharide, which consists of randomly arranged units of glucosamine and N-acetylglucosamine linked by a β-1,4 bond, is derived from the biopolymer chitin by a deacetylation process. As a polysaccharide, it provides an abundance of hydroxyl (OH) groups with excellent ability to H-bond during the drying process, allowing the formation of microspheres, fibers, films, and membranes. CS is weakly alkaline due to the glucosamine monomer containing a primary NH_2_ group with a pKa of about 6.5 [11], which is protonated at low pH values, promoting solubility and film formation of CS in acidic solutions and stability at neutral to high pH values. The latter makes it highly resistant in alkali-rich media, such as those found in AEMFCs. However, its brittleness requires blending with another polymer or doping with fillers to reduce cracking and improve handling. The ionic conductivity, fuel permeability, and ion exchange capacity, as well as the mechanical and thermal properties of AEMs, can be improved by modifying biopolymers, or by introducing fillers and forming composites [12]. To improve the properties of biopolymer membranes, various fillers have been incorporated into the chitosan matrix, including multi-walled carbon nanotubes [13], functionalized MXene [14], N-doped graphene oxide [14,15], and a composite of quaternized cellulose nanocrystals and quaternized PPO [16].

In our previous work [15,17,18], we demonstrated the ability of CS membranes with modified graphene oxide (GO) as inorganic fillers to improve the cell performance of AEMFCs. In this article, we use the organosilane reagent to modify cellulose nanofibrils (CNFs), and use them as quaternary ammonium-bearing fillers, which is the novelty of this work. We hypothesize that the combination of CS with (modified) CNF fillers will bring numerous advantages to CS membranes in terms of mechanical and thermal enhancement, ionic conduction, and, ultimately, an increase in power density while preventing EtOH crossover. With extensive physicochemical and morphological characterization, we seek to elucidate the effects of the CNF filler and its modified counterpart on composite properties and overall performance in a laboratory-scale fuel cell assembly.

## 2. Materials and Methods

### 2.1. Materials

Chitosan (CS, degree of deacetylation: 90%, molecular weight: 50–100 kDa, particle size < 200 µm) was purchased from Biolog Heppe GmbH, Landsberg, Germany. Nanofibrillated cellulose (CNF, ~3 wt.% water dispersion) was purchased from the University of Maine, Orono, ME, USA. Dimethyloctadecyl[3-(trimethoxysilyl)propyl]ammonium chloride (DMAOP, 42 wt.% solution in methanol), magnesium hydroxide Mg(OH)_2_ nanopowder (<100 nm particle size (laser PSA), 99.8% trace metals basis), sodium hydroxide (NaOH), hydrochloric acid (HCl), ethanol (EtOH), and potassium hydroxide (KOH) were purchased from and manufactured by Sigma Aldrich, Darmstadt, Germany, and used without further purification. All the reagents and solvents were analytical grade and used as received. The Fumasep Fumatech FAA-3-50 membrane was obtained from Fumatech BWT GmbH, Bietigheim-Bissingen, Germany.

### 2.2. CNF Quaternization

Modification of CNF by the DMAOP reagent was performed according to the following procedure: component A (10 g 3 wt.% CNF in 40 mL methanol, 30 min mixing, room temperature) was mixed with component B (25 mL DMAOP reagent was added to 100 mL Milli-Q water, 2 h mixing, room temperature, pH adjusted to 2, 4, 6, 8, or 10). The resulting mixtures were stirred additionally for 24 h, and the final dispersion was centrifuged (9000 min^−1^, 10 min), and the precipitate was washed thoroughly with Milli-Q water by centrifugation at 9000 min^−1^, freeze dried, and, finally, heated in an oven at 110, 130, or 150 °C for 1, 2, or 3 h. The resulting modified CNF was used as the final product and designated CNF(D).

### 2.3. Characterization of CNF

The attenuated total reflectance-Fourier transform infrared spectroscopy (ATR-FTIR) spectra were recorded using a Perkin-Elmer Spectrum One FTIR spectrometer (Waltham, MA, USA) with a Golden Gate ATR attachment and a diamond crystal. The transmission spectra were recorded in the range 4000 to 650 cm^−1^. Each spectrum obtained is the average of 16 spectra recorded at a resolution of 4 cm^−1^. The Nuclear Magnetic Resonance (NMR) spectra were recorded using the NMR recording technique on solid samples on a Bruker AVANCE NEO 400 MHz NMR spectrometer (Bruker, Billerica, MA, USA) with a 4 mm CP-MAS probe and TopSpin 4.0.9 software. The Larmor frequencies of the 13C and 29Si nuclei were 100.63 and 79.49 MHz, respectively. The chemical shifts of the 13C and 29Si NMR nuclei are given relative to the TMS Standard (δ 0.0 ppm). The samples were rotated at 15,000 Hz for all measurements. The zeta potential (ζ) was determined by the dynamic light scattering (DLS) method at 25 °C at a 90° angle with a Zetasizer Nano ZS90 (Malvern Panalytical Ltd., Worcestershire, United Kingdom) system using capillary cells. The combined TGA-DSC analysis was performed using a Mettler Toledo Thermogravimetric Analyser/Differential Scanning Calorimeter (TGA/DSC1) (Mettler-Toledo International Inc., Greifensee, Switzerland) thermal analyzer. The test temperature range was between 30 and 700 °C with a heating step of 10 K/min under an N_2_ atmosphere. The morphology of the CNF was imaged by Field Emission Scanning Electron Microscopy (FE-SEM) on a Carl Zeiss FE-SEM SUPRA 35 VP electron microscope (Carl Zeiss, Yena, Germany) at a 1 kV accelerating voltage and a working distance of about 4.5 mm. The samples were mounted on an aluminum support and sputtered with a 10 nm thick palladium layer.

### 2.4. Membrane Preparation

The neat CS membranes were prepared as described previously [17,19]. Briefly, the CS was dissolved in Milli-Q water, while the pH was adjusted to 2 (with 1 M HCl), and a 1% wt.% aqueous Mg(OH)_2_ dispersion was added until a pH of 6 was reached. The obtained dispersion was diluted to the final amount of 1 wt.% CS and 12.6 mM Mg(OH)_2_, and 25 mL was poured into a Petri dish with a diameter of 8.5 cm. After drying in an oven at 60 °C for 24 h, the membrane was neutralized in 1 M NaOH with shaking for 30 min at room temperature, then washed with Milli-Q water and air dried. For the preparation of the CS-CNF composite membranes, the selected amounts of CNF or CNF(D) fillers were added to a CS-Mg (OH)_2_ dispersion. The names of the membranes prepared with the corresponding ingredients are listed below in Table 1.

### 2.5. Membrane Characterization

The ATR-FTIR spectra were recorded in the same manner as for the CNF fillers. The XRD spectra of the membranes were recorded using a Bruker Siemens D5005 X-ray diffractometer (Bruker, Billerica, MA, USA). The reflections at 2θ were observed in the range of 2° to 70°, with an increment of 0.04° when using Cu-Kα radiation at a voltage of 40 kV and a current of 40 mA. The relative degree of crystallinity of the polymer phase was determined from the ratio between the integrated area of the crystal peaks and the total integrated area of the diffraction spectrum [20]. SEM imaging was performed using an FEI Sirion 400 NC (Sirion 400 NC, FEI, Hillsboro, OR, USA) with an INCA 350 EDS electron microscope with 9 kV accelerating voltage from a working distance of about 5–6.9 mm. Prior to analysis, the samples were sputtered with a 10 nm thick gold layer. The thermal stability of the membranes was determined by TGA analysis using a Perkin-Elmer TGA 8000 thermogravimetric analyzer (Waltham, MA, USA). The temperature range of the test was between 30 °C and 700 °C, with a heating step of 10 K/min in a N_2_ atmosphere. The mechanical properties of the membranes were determined by tensile testing using the Shimadzu Europa GmbH AG-X plus 10 kN instrument (Düsseldorf, Germany). The membrane samples were cut into a rectangular shape of 1 × 2 cm, the distance between the clamps was 20 mm, and the tension speed was set to 1 mm/min. The passage of EtOH through the CS membranes was measured in temperature-controlled diffusion cells at 25 ± 1 °C [17,18]. The diffusion cells consisted of two glass chambers (a chamber with a solution source—chamber A and a receiving chamber—chamber B). Each of the chambers holds 25 mL of solution, and between the chambers is a membrane in a plastic holder separating the chambers. Before each experiment, the membrane was soaked in distilled water for 24 h to avoid the influence of base release from the membrane to the solution. Compartment A was filled with 25 mL of 2 M EtOH in a 6 M KOH solution. Compartment B was filled with 25 mL of 6 M KOH solution. The membrane sample had an effective surface area of 7.03 cm^2^. The concentration of EtOH flowing through was determined by conductivity measurements taken with a conductometer in chamber B at different time intervals. The EtOH transfer, *P* (cm^2^ s^−1^), was calculated according to Equation (1):

(1) $$P\left(\frac{cm^{2}}{s}\right)=\frac{\left(C_{B}-C_{B0}\right)}{\left(t-t_{0}\right)}\cdot \frac{V_{B}\cdot l}{A\cdot C_{A0}}$$

where *C*_*A*0_ is the initial concentration of EtOH in the solution source compartment (compartment A), and *V_B_* is the KOH volume in the receiving compartment (compartment B), A is the area of the membrane sample, and *l* is the thickness of the membrane sample. The KOH uptake and swelling ratio tests of the CS membranes were performed in 6 M KOH at 60 °C. The dry membranes were cut to a size of 1 cm × 1 cm and weighed, and their mass (*W_dry_*), surface area (*A_dry_*), and thickness (*T_dry_*) were recorded. The membranes were then immersed in an alkaline medium, 6 M KOH, at 60 °C. After immersion, the excess alkali solution on the surface of the membranes was removed with a paper towel and weighed (*Wwet*), and their dimension-based surface area (*A_wet_*) and thickness (*T_wet_*) were measured at room temperature. The alkali uptake (*AU*) and through-plane (*SR_Tp_*) and in-plane (*SR_Ip_*) swelling ratios were determined after 24 h using the following equations:

(2) $$AU=\frac{W_{wet}-W_{dry}}{W_{dry}}\;\cdot 100\;\%$$

(3) $$SR_{Through-plane}=\frac{T_{wet}-T_{dry}}{T_{dry}}\;\cdot 100\;\%$$

(4) $$SR_{In-plane}=\frac{A_{wet}-A_{dry}}{A_{dry}}\;\cdot 100\;\%$$

The ion exchange capacity (*IEC*) values were determined by potentiometric titration. The anion exchange membranes in OH^−^ form (pre-dipped in 1 M NaOH) were then immersed in 40 mL of 0.01 M HCl for 24 h. These solutions were then titrated with a standard solution of 0.1 M KOH. Before immersing the membrane and performing the titration, we first cut and weighed about 0.02 g of membrane sample and noted its exact mass. After titration, the IEC parameter was calculated, and expressed as the milliequivalent (meq) of OH^−^ ions per gram of dry membrane using the following Equation (5):

(5) $$IEC\left(\frac{meq}{g}\right)=\frac{\left(V_{blank}-V_{membrane}\right)\cdot c_{HCl}}{m_{dry\;membrane}}$$

where *V_blank_* and *V_membrane_* represent the volumes (mL) of 0.1 M KOH solution consumed for the blank and sample membranes, respectively. *c_HCl_* represents the molar concentration (mol/L) of the HCl solution and *m_dr_*_y membrane_ represents the mass of the dry membrane samples (g). The ionic conductivity of the membranes was measured in Milli-Q water in the frequency range from 125 Hz to 10^6^ Hz using two-electrode AC impedance spectroscopy, which was used to analyze the impedance response of the membrane sample and to determine the resistance. Prior to the measurement, the membrane sample was cut to a size of 1 × 3 cm^2^ and soaked in Milli-Q water for 24 h. The ionic conductivity of the membranes was calculated using Equation (6):

(6) $$\sigma \left(\frac{mS}{cm}\right)=\frac{L}{R\;\cdot S}$$

where *L* (cm) is the distance between the two electrodes, and *S* (cm^2^) is the area (28.28 mm^2^) of the sample subjected to measurements. The resistance of the membrane is expressed by *R* (Ω).

### 2.6. Cell Performance Test

The cell performance test for the obtained membranes and the reference membrane Fumatech FAA-3-50 was performed in an optimized test setup [21] as follows. The membranes were first immersed in 1 M KOH for 24 h and then washed thoroughly with ultrapure water to remove the excess KOH. The electrodes were prepared by depositing the ink from the anode/cathode catalysts in 2-propanol (99.9%) and ultrapure water (7:3) and a commercial ionomer onto the GDL using a Sono-Tek ultrasonic spray coater. The cathode was prepared by spraying a commercial PtRu/C catalyst (platinum 40%, ruthenium 20% on carbon black, HiSPEC^®^ 10000) on carbon paper (Sigracet 29 BC, fuel cell store, 0.235 mm thick), and the PdNiBi/C catalyst ink [22] was applied on carbon cloth (ELAT-hydrophobic plain cloth, fuel cell store, 0.406 mm thick), which constituted the anode. Electrodes with a cathode metal loading of 0.5 mg cm^−2^ and an anode metal loading of 0.75 mg cm^−2^ were obtained with this preparation. The MEA consisted of the mentioned electrodes and the membrane, a sample of CS and (un)modified CNF or the commercial membrane, and was used in a self-designed alkaline direct ethanol fuel cell [21]. The operating temperature and concentration of the supplied fuel were varied. Pure oxygen was fed to the fuel cell at a constant flow of 25 mL min^−1^ at the cathode, and a mixture of 1 M ethanol and 1 M KOH or a mixture of 3 M EtOH and 5 M KOH with a constant flow of 5 mL min^−1^ at the anode. The measurements were performed at room temperature, 60 °C and 80 °C. The polarization curves (I-V diagram) were obtained using a Zahner IM6ex potentiostat. The results were plotted as an I-V diagram with additional representation of the power density.

## 3. Results and Discussion

### 3.1. CNF Quaternization

**Figure 1:**
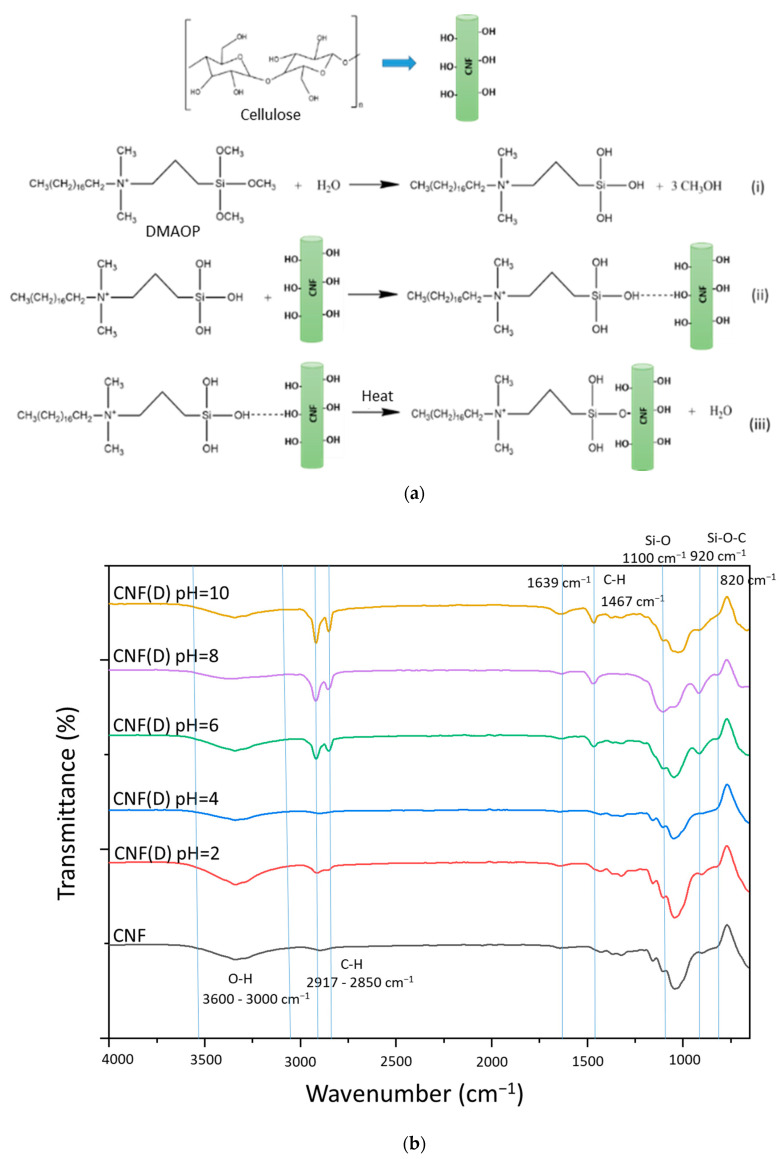
(**a**) Tentative representation of the reaction between cellulose nanofibrils (CNF) and the Dimethyloctadecyl[3-(trimethoxysilyl)propyl]ammonium chloride (DMAOP) reagent and (**b**) Attenuated total reflectance-Fourier transform infrared spectroscopy (ATR-FTIR) spectral lines for the reference (CNF) and modified quaternized CNFs (CNF(D)) at the pH 2–10.

The first step of the reaction between CNF and the silane reagent DMAOP in the presence of water consists of the hydrolysis of the alkoxy groups of the latter to form silanols (Figure 1a). The acid-catalyzed hydrolysis of silanes allows the formation of silanol groups, reduces self-condensation reactions between the silanol groups, and keeps the hydrolyzed intermediates stable. In contrast, under basic conditions, the condensation reaction begins as soon as the hydrolysis reaction of silanes starts, leading to rapid consumption of silanol groups by the self-condensation reaction, and the formation of three-dimensional structures with high M_w_ [23].

To demonstrate the pH dependence of the modification process, the ATR-FTIR spectral lines (Figure 1b) for neat and modified CNF were examined at different pH values. The spectral line for the reference CNF shows a broad band at 3600–3000 cm^−1^ (O-H stretching) and ~2900 cm^−1^ (symmetric and asymmetric C-H stretching), 1000–1070 cm^−1^ is the region of primary (~1030 cm^−1^) and secondary (~1055 cm^−1^) alcohols, and the small band ~900 cm^−1^ was assigned to ether (C-O-C stretching) [24]. The pronounced band at ~2850 cm^−1^ appeared in the samples after the reaction with DMAOP and is associated with the presence of a long nonpolar alkyl chain on the reagent. The weak peak at 1639 cm^−1^ illustrates the vibrations of the remaining absorbed water [25]. The increased intensity of this peak, especially at high pH, is consistent with the Si-OH form of the DMAOP reagent, and suggests its interaction with the OH groups on cellulose via H-bonds [26]. The peak at a wavelength of about 1467 cm^−1^ is associated with the quaternary ammonium group in the DMAOP molecule, and can also be assigned to the C-H bond, illustrating the bending of the alkyl groups attached to the quaternary ammonium [25,27,28,29]. The characteristic peaks for Si-O alkoxysilanes can be observed at about 1090 cm^−1^ [30], and the peaks at about 1100 and 820 cm^−1^ show the vibrations of symmetric and asymmetric Si-O-Si stretching [31]. The band at 1820 cm^−1^ is observed in the products at reactions pH = 6 and pH = 8, indicating that the DMAOP reagent has self-polymerized and formed Si-O-Si bonds. The peaks in the 950–700 cm^−1^ range consisted of Si-C, C-O, and Si-O bonds, and, in combination, these peaks represent the Si-O-C bond [32]. The peak at about 920 cm^−1^ is most prominent in the samples obtained at pH = 6 and pH = 8, indicating the formation of Si-O-C bonds between the CNF and DMAOP.

**Figure 2:**
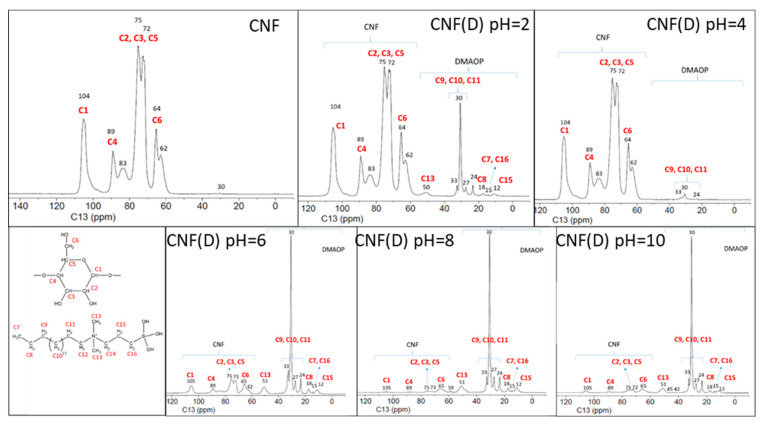
Carbon-13 (C13) nuclear magnetic resonance (^13^C NMR) spectra of reference CNF and CNF(D) at different pH reaction conditions (2, 4, 6, 8 and 10).

The ^13^C NMR data of reference CNF (Figure 2) show a resonance signal between 62 and 104 ppm, which can be attributed to the different forms of C atoms in the cellulose monomer unit (C1-C6).

**Figure 3:**
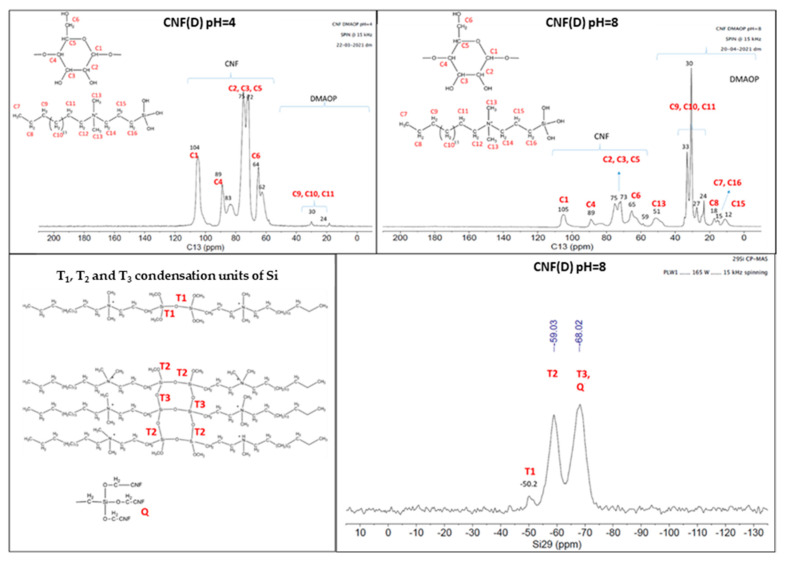
^13^C NMR and ^29^Si NMR spectra of CNF(D) samples at pH = 4 and pH = 8 reaction conditions.

The spectra of the modified (CNF(D)) samples obtained at different pH conditions show additional resonance signals in the range of 12 to 33 ppm, which can be attributed to C atoms in the DMAOP reagent (C7-C16). It should be emphasized that the analysis was performed after intensive repeated washing of the reaction products with water by centrifugation, which presumably prevents the presence of the DMAOP reagent in free form. Moreover, the peaks characteristic of the C atoms in the DMAOP reagent are more pronounced for the product at reactions pH = 6, pH = 8, and pH = 10, while the peaks for the product obtained under acidic conditions are less pronounced, especially at pH = 4, where only a fleeting signal is observed for the three carbon atoms present in the DMAOP. Based on the NMR results, we could not determine the type of binding (electrostatic/covalent) of DMAOP to CNF. Next, we performed additional washing with ethanol, and again recorded the ^13^C NMR spectra of the samples prepared at pH = 4 and pH = 8 (Figure 3), as they differed the most. Therefore, we were interested in whether the CNF(D) sample was stable after washing with ethanol, which is present in ethanol fuel cells, thus providing a modified CNF that is stable under fuel cell operating conditions. The detected carbon signals in the DMAOP reagent between 12 ppm and 52 ppm on the CNF product (reaction pH = 8) [33] indicated persistent binding between the CNF and DMAOP at this pH.

Examination of the ^13^C NMR spectra for the sample after the reaction between the CNF and DMAOP at reaction conditions of pH = 4 and pH = 8 after extensive washing of the product with ethanol and water revealed that the reagent DMAOP was absent in the product obtained in the reaction of pH = 4, while it was readily detectable in the product obtained at the reaction of pH = 8. The ^29^Si NMR spectrum (Figure 3) for the sample CNF(D) pH = 8 gives us information about the bonding of the Si atoms in the product, and we observed three different resonance signals between −50 ppm and −68 ppm due to different Si units formed during the condensation of the reagent.

**Figure 4:**
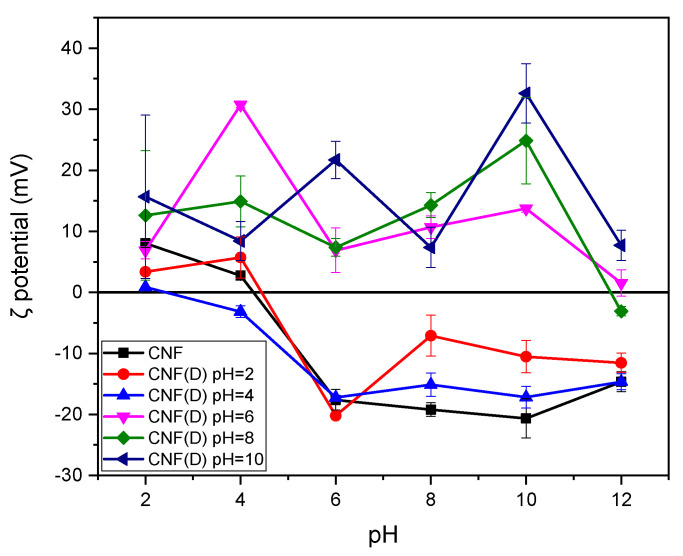
ζ potential values (mV) for CNF and CNF(D) at different pH reaction conditions (2, 4, 6, 8, and 10).

The presence of a positive charge on the modified CNF was investigated further based on the ζ-potential measured in the pH range of 2–12 (Figure 4). CNF itself has an isoelectric point at a low pH (~4), with negative ζ-potential values at a pH of >4 due to deprotonation of the carboxyl group of the remaining hemicellulose residue from CNF processing [34,35]. The positive charge of the CNF(D) samples was caused by the quaternary ammonium group of the DMAOP reagent, as this group carries a permanent positive charge. Thus, the results confirmed the presence of a permanent positive charge in the reaction product obtained at pH = 6 to pH = 10, as evidenced by the ATR-FTIR and NMR data.

**Figure 5:**
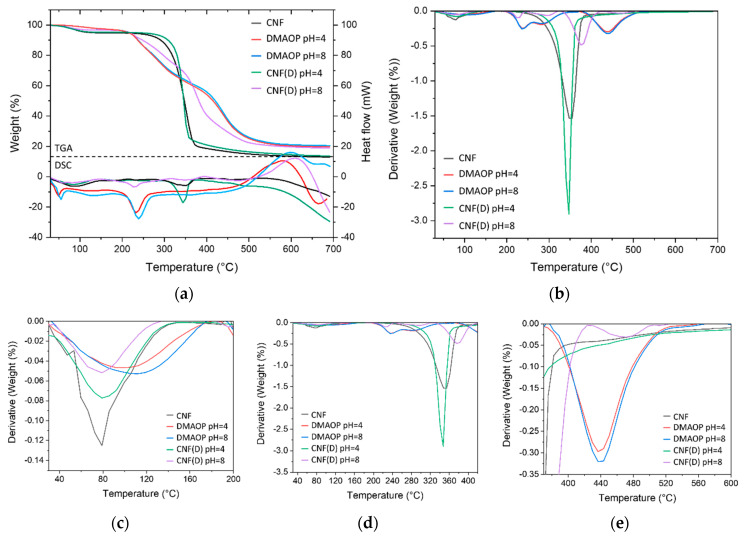
(**a**) Thermogravimetric Analysis (TGA) and Differential Scanning Calorimetry (DSC) curves, (**b**) derivation of the TGA curve with approximate areas (**c**–**e**), for samples of the reference CNF, the hydrolyzed reagent DMAOP at pH = 4 and pH = 8, and the product CNF(D) at pH = 4 and pH = 8.

The temperature stability of the CNF(D) products was evaluated by TGA and DSC analysis (Figure 5, Table 2). For all the samples tested, the observed mass loss in the temperature range from 30 to 180 °C was due to evaporation of the absorbed water. The degradation of CNF typically occurred in a single step in the range of 245–380 °C with a mass loss of about 75% [36], while the degradation of the DMAOP reagent occurred in two steps, namely, in the range of 205–265 °C with a mass loss of 14% associated with the decomposition of the quaternary ammonium groups, and in the range of 370–525 °C with a mass loss of 37% when pyrolysis occurred of the long, nonpolar alkyl chain [37]. For the CNF(D) pH = 4 sample, one-step degradation was observed in the range of 255–370 °C with a mass loss of 71%, which is consistent with the CNF sample, and indicates unsuccessful modification. The CNF(D) pH = 8 sample showed three mass loss zones, representing a one-step degradation of the CNF component in the 330–420 °C range, with 37% mass loss and a two-step degradation of the DMAOP component in the 205–245 °C range with 11% mass loss, and in the 430–510 °C range with 12% mass loss. Moreover, the CNF(D) sample obtained at pH = 8 degraded at a higher temperature than the pure CNF reference sample, which is a clear indication of improved stability after modification. In the DSC thermogram of the neat CNF sample, an endothermic peak can be seen at about 350 °C, which can be attributed to the degradation of CNF due to the breaking of glycosidic bonds, leading to the depolymerization of the cellulose. In comparison, the CNF(D) pH = 8 sample showed an additional endothermic peak at about 230 °C, which was also observed for the DMAOP pH = 4 and DMAOP pH = 8 samples and belongs to the DMAOP reagent, representing the decomposition of the silane [38] and disintegration of the DMAOP polymer network (breaking of the Si-O-Si and S-O-C bonds). The indicated transition was not observed in the CNF(D) pH = 4 and CNF reference samples, confirming the previous findings for unsuccessful modification.

**Figure 6:**
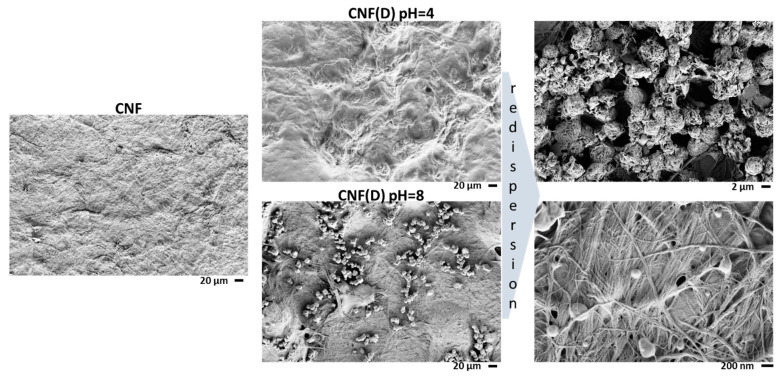
Field Emission Scanning Electron Microscopy (FE-SEM) images of CNF and CNF(D) at pH = 4 and pH = 8 reaction conditions before and after redispersion in Milli-Q water.

The effect of DMAOP modification on CNF morphology was examined using FE-SEM imaging (Figure 6). A large heterogeneity of the CNF sample was observed, as well as the presence of aggregates formed by intense hydrogen bonding during the drying process, grouping CNF fibrils with a fiber diameter of ~50 nm and a length of 100 µm into larger clusters. In the image of the CNF(D) pH = 8 product, particles of about 10 µm can be seen that are absent in the CNF(D) pH = 4 product, and we assumed that these are aggregates of the DMAOP reagent. As mentioned earlier, the hydrolysis of the DMAOP reagent is slower under acidic conditions than under alkaline conditions. This is why, at pH = 8, the DMAOP reagent not only reacts with cellulose but also polymerizes with itself to form aggregates of the polymerized DMAOP reagent, which explains their detection on the SEM images. Since the manufacturing process of the CNF(D) product involves a drying step, redispersion is required when this product is incorporated into membranes. Redispersion is somewhat limited, due to the H-bonds formed between the CNF(D) product. In the SEM images of redispersed CNF(D) pH = 8, particles can still be seen that are presumably polymerized clumps of the DMAOP reagent.

Based on the results, the efficient modification of CNF by the DMAOP reagent was confirmed for the pH = 8 reaction condition, so we selected the CNF(D) pH = 8 sample as the optimal product and included it in further CS membranes.

### 3.2. Characterization of CS Membranes: Effect of Fillers on Membrane Properties

**Figure 7:**
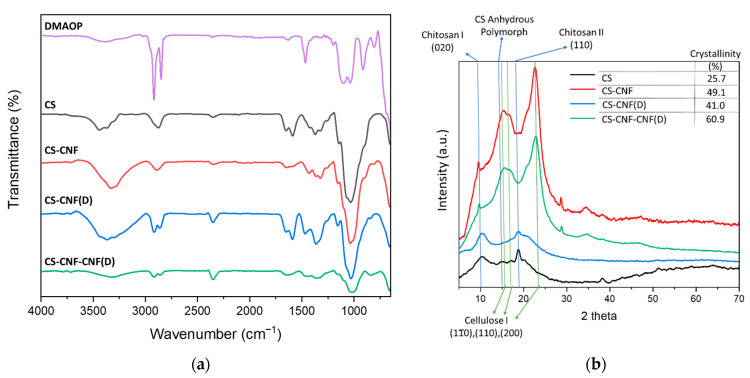
(**a**) ATR-FTIR spectral lines of CS membranes, including CNF(D) and corresponding reference membranes, and (**b**) X-ray diffractograms (XRD) of CS membranes containing CNF and CNF(D) fillers.

Figure 7a shows the collected ATR-FTIR spectra of CS membranes with CNF and CNF(D) fillers. In addition to the peaks attributed to CNF and CNF(D) fillers, as described in Section 3.1 CNF Quaternization, observed peaks were attributed to the CS polymer: a broad peak from 3650–3250 cm^−1^ attributed to O-H stretching vibrations, with three well-defined peaks attributed to free OH groups (3450 cm^−1^), N-H stretching (3370 cm^−1^), and H-bonded O-H stretching (3300 cm^−1^); the peak at ~2900 cm^−1^ was attributed to C-H stretching, that at 1647 cm^−1^ to C=O vibrations in the amide group, that at 1590 cm^−1^ to N-H vibrations, that at 1144 cm^−1^ to asymmetric stretching of the C-O-C bridge, and that at 1150 cm^−1^ to the skeletal vibration involving C-O stretching [19].

In the case of the neat CS, as well as the CS-CNF membrane, the peak at 2900 cm^−1^ is single, and attributed to the C-H stretching of the atoms in the polysaccharide matrix, and in the case of the presence of CNF(D) in the CS composite membrane, a double sharp peak occurred, which was attributed to the long nonpolar tail of the DMAOP molecule, confirming the presence of modified CNF in the CS membranes.

Figure 7b shows the XRD diffractograms of the neat CS and the membranes with (modified) CNF as a filler. Since all the membranes contained CS, sharp peaks were observed in all the samples at about 2θ = 10° (020) and 2θ = 19° (110), which are characteristic of the crystal structure of the hydrated crystal of CS I and the anhydrous CS II, respectively. The broad peak between 2θ = 13° and 19° was due to the amorphous structure of the CS membrane. The calculated crystallinity index of the neat CS membrane was 25.7%. By adding fillers, we were able to increase the crystallinity index of the CS membranes. Zhang et al. [39] reported an increase in the crystallinity of CS membranes with increasing the filler content of the modified TEMPO-CNF from 22% for CS membranes to 87%. We observed an approximately twofold increase in the crystallinity of CS membranes when CNF fillers were added, and an additional increase (up to 60.9%) when a mixture of CNF and CNF(D) fillers was added, indicating a compatibilization of the fillers with the CS matrix, due mainly to H-bonds between the two polysaccharide components. For all CS membranes containing CNF, the XRD pattern of crystalline cellulose polymorph I is observed with diffraction peaks at 2θ at about 15°, 17°, and 23.5°, belonging to the (110), (110), and (200) planes, respectively (Gong et al. 2017). For the CS-CNF (D) sample, which contained only the modified CNF(D) in addition to CS, we did not observe the diffraction peaks characteristic of cellulose I, but a broad amorphous peak between 15° and 25°, assuming that the DMAOP treatment affects the crystallinity of the CNF itself.

**Figure 8:**
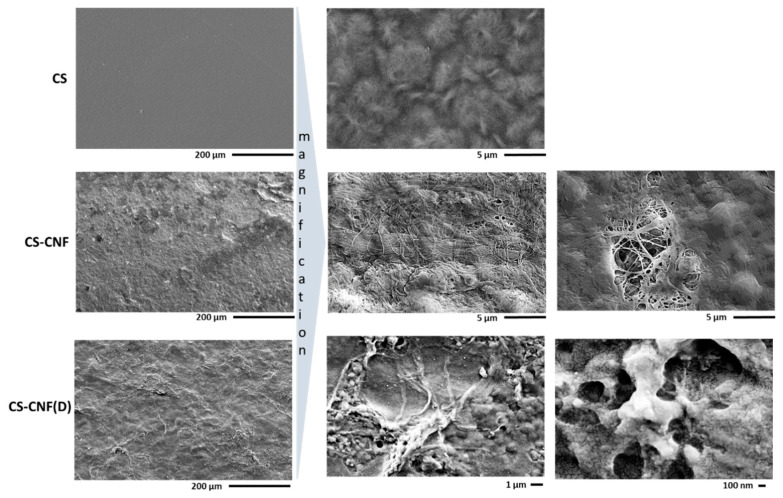
FE-SEM images of CS membranes with CNF and CNF(D) fillers.

Figure 8 shows an SEM image of the homogeneous and smooth CS membrane with no visible pores, whereas the CS-CNF membrane contains both agglomerates of CNF fibers and individual intertwined fibers, and also has desiccation cracks on the top surface that allow a view into the depth and reveal a network of intertwined CNF fibers. The membrane CS-CNF (D) shows a strong agglomeration and non-uniform distribution of the CNF(D) filler in the CS matrix of the membrane, as well as clusters (< µm in size) on the fibers, which are presumably the polymerized reagent DMAOP, as also observed by SEM analysis of the CNF(D) fillers themselves (Figure 6).

### 3.3. Thermal Stability of the Membranes

**Figure 9:**
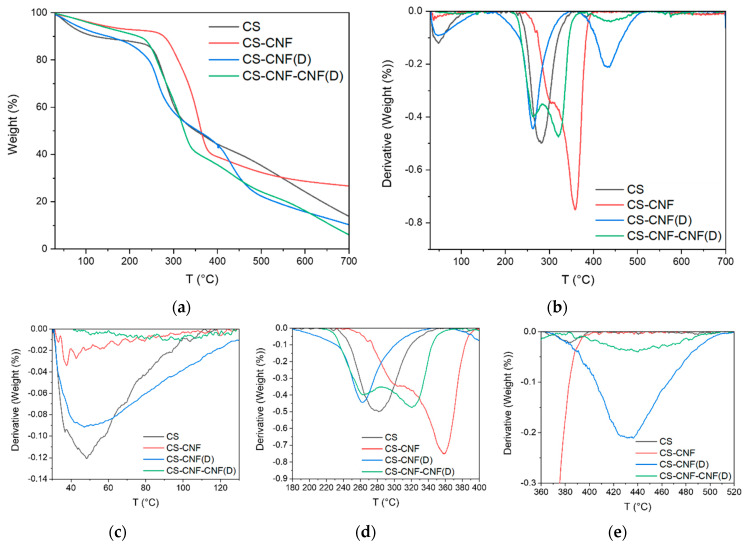
(**a**) TGA curves, (**b**) the first derivative of TGA curve, (**c**) display of the first mass loss area, (**d**) display of the second mass loss area, and (**e**) display of the third mass loss area for CS membranes with CNF and CNF(D) fillers.

Examining the thermographs (Figure 9, Table 3) of CS membranes with CNF and CNF(D) fillers, we can see three ranges of mass loss, namely, 30–135 °C (T1) due to water evaporation; 180–400 °C (T2) due to the degradation of the polymer network in CS, CNF, and modified CNF(D) components, including the degradation of glycosidic bonds and glucopyranose units [40]; and 360–520 °C (T3), due to the degradation of the CNF(D) filler. The addition of fillers to the CS membrane leads to a change in the initial temperature of degradation (T_s_) and the peak temperature of degradation (T_p_), as well as a change in mass loss. The addition of neat CNF into the CS membrane resulted in a 15% lower mass loss over the entire temperature range from 30 to 700 °C (∆y) compared to the reference CS membrane. The addition of the CNF filler improved the thermal stability of the CS membranes, as we found that the membranes with this filler reached the peak of thermal decomposition at a higher temperature (300–305 °C) than the reference CS membrane (283 °C). Compared to the CS membrane, the total mass loss of the membranes with this filler also decreased (from 85.7% in CS to 72.6% in CS-CNF). The addition of the CNF(D) filler led to a 4% increase in mass loss, which can be attributed to the instability of the quaternary ammonium groups of the modified filler. As with the CNF(D) filler itself (Figure 5), we also observed an additional range of mass loss at T3_p_ around 430 °C for CS membranes with CNF(D) filler, which represents the degradation of the CNF(D) filler. The thermal stability of polymers is related to their degree of crystallinity, and usually a higher degree of crystallinity leads to better thermal stability. The CS-CNF and CS-CNF-CNF (D) membranes were found to have higher crystallinity compared to the reference CS membrane, which explains the higher degradation temperature peaks for these membranes.

### 3.4. Mechanical Properties, Swelling Ratio, Alkaline Uptake, Ethanol Permeability, IEC and Ion Conductivity of the CS Composite Membranes

**Figure 10:**
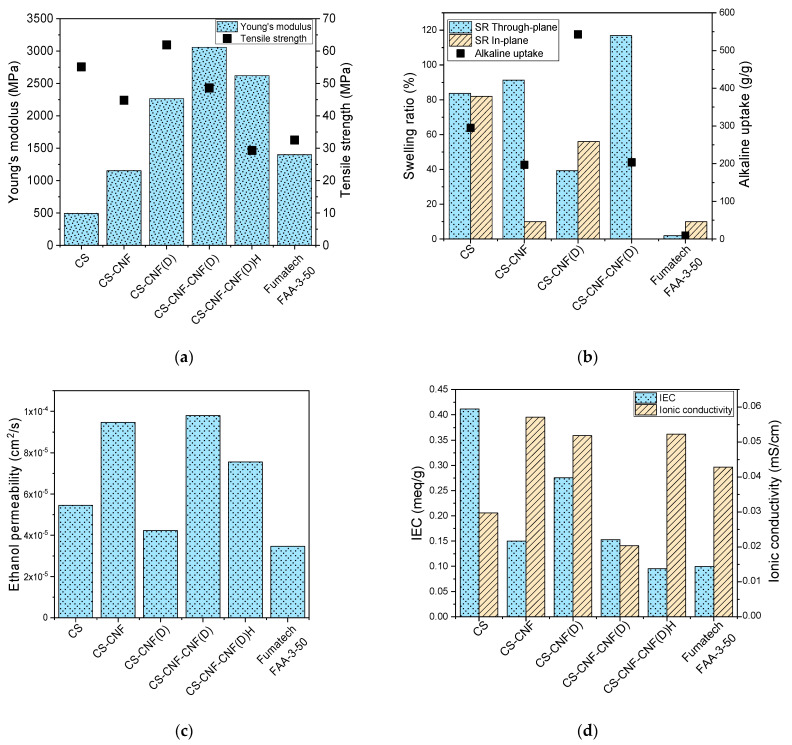
Comparison of the membrane’s (**a**) mechanical properties, (**b**) swelling ratio and alkaline uptake, (**c**) ethanol (EtOH) permeability, (**d**) ion exchange capacity and ionic conductivity for the CS-based AEMs and the commercial Fumatech FAA-3-50 AEM.

Figure 10a shows that the mechanical properties of the CS-based AEMs are comparable to, or even better than, those of the commercial Fumatech FAA-3-50 membrane. We observed excellent mechanical properties of the CS-CNF (D) membrane with a Young’s modulus of 2.26 GPa and a tensile strength of 61.9 MPa. Compared to the reference CS membrane, a strong improvement in Young’s modulus was observed for all membranes with functionalized CNF filler (359% for CS-CNF (D), 520% for CS-CNF-CNF (D), 431% for CS-CNF-CNF (D)H), indicating the increased degree of crystallinity of these membranes compared to the reference CS membrane. Compared to the reference CS membrane, there was a 12% improvement in tensile strength for the CS-CNF (D) membrane.

Membrane swelling and alkali uptake are shown in Figure 10b. A significant increase in through-plane swelling and alkali uptake was observed for the CS-based membranes with CNF fillers compared to the Fumatech FAA-3-50. There was a correlation between the higher alkali uptake and higher ionic conductivity [16], and we also observed an increase in the alkali uptake of the CS-based membranes compared to the Fumatech FAA-3-50, as well as increased ionic conductivity for some of the CS-based membranes compared to the Fumatech FAA-3-50. Through-plane swelling can strengthen the contact between the current collectors and the membrane electrode assembly in the AAEMFC, which has a positive effect on the operation of the fuel cell. Conversely, in-plane swelling can loosen the contact between the AEM and the catalysts, and increase the resistance of the membrane electrode assembly. [41] For this reason, it is desirable that in-plane swelling be as low as possible, as this is the only way to ensure that the dimensional stability of the membrane is constant during fuel cell operation. Following these findings, we observed the highest power density for the membrane (CS-CNF) that also had the largest through-plane and low in-plane swelling. Accordingly, the CS-CNF-CNF (D) membrane with the highest through-plane swelling ratio and the lowest in-plane swelling provided the highest power density of the CS--based membranes with CNF(D) fillers.

For AEMFC applications, the fuel permeability of the membrane should be kept as low as possible, because high fuel transfer leads to reduced fuel cell efficiency. Figure 10c shows that the CNF(D)-containing membrane had the lowest ethanol permeability (4.23 × 10^−5^ cm^2^ s^−1^) of the CS-based AEMs compared to all the manufactured CS-based membranes, while the value for the commercial Fumatech FAA-3-50 membrane was in the same range (3.47 × 10^−5^ cm^2^ s^−1^).

Figure 10d shows the IEC and ionic conductivity properties of the AEMs. For all CS-based membranes with fillers, except CS-CNF (D), an improvement in ionic conductivity was observed compared to the commercial membrane, while the reference CS membrane had a lower ionic conductivity than the commercial membrane. The reason for the improvement in the ionic conductivity of the membranes with fillers, but not in that of the reference CS membrane, is that the presence of functionalized CNF fillers introduces a positive charge into the membrane. In addition, the presence of CNF-based fillers allows for a more diverse three-dimensional structure of the membranes, and can lead to the formation of a network of intertwined fibers with gaps present, which contributes to the transfer of OH-ions through the membrane, since ionic conduction by the diffusion mechanism occurs only when free volume is available within the polymer chains. The reference CS membrane exhibited the highest IEC (0.41 meq/g), followed by the CS-CNF (D) membrane (0.28 meq/g), which had a higher IEC compared to the CS-CNF membrane with unmodified filler (0.15 meq/g), due to the presence of quaternary ammonium groups on CNF(D).

### 3.5. Direct Ethanol Alkaline Fuel Cell (DEAFCs) Performance

**Figure 11:**
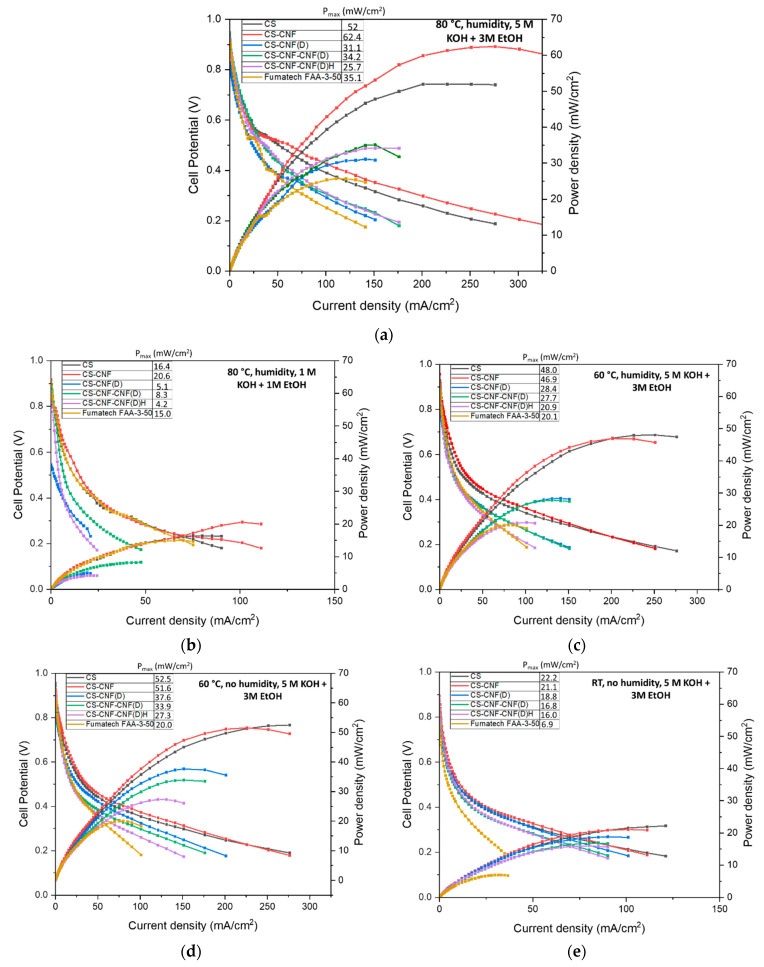
The results of membrane testing in the alkaline anion exchange membrane fuel cells (AAEMFC) under different operating conditions. Cell voltage and power density versus current density plots using CS membranes with or without CNF and CNF(D) fillers and a commercial Fumatech FAA-3-50 under different operating conditions: (**a**) 80 °C, humidified oxygen, and a fuel mixture of 5 M KOH and 3 M EtOH, (**b**) 80 °C, humidified oxygen, and a fuel mixture of 1 M KOH and 1 M EtOH, (**c**) 60 °C, humidified oxygen, and a fuel mixture of 5 M KOH and 3 M EtOH, (**d**) 60 °C, without oxygen humidification, and a fuel mixture of 5 M KOH and 3 M EtOH, (**e**) RT, without oxygen humidification, and a fuel mixture of 5 M KOH and 3 M EtOH.

The prepared CS membranes with CNF and CNF(D) fillers were tested in an ethanol fuel cell under different operating conditions, and compared with the commercial Fumatech FAA-3-50 membrane (Figure 11a–e). The presence of humidified oxygen instead of non-humidified oxygen had almost no effect on the commercial Fumatech FAA-3-50 membrane; on the contrary, for all CS membranes, the presence of humidified oxygen at the same temperature (60 °C) resulted in a decrease in P_max_ compared to the fuel cell in which non-humidified oxygen was fed at the cathode. We attributed this to the highly hydrophilic nature of the CS-based membranes, which resulted in higher water uptake, which, if too high, leads to reduced fuel cell efficiency. For fuel cells operating at room temperature with non-humidified oxygen, we found that, compared to the reference CS membrane (22.2 mW cm^−2^), the CS-CNF (D) membrane had a lower power density at all operating conditions of the fuel cell (15–70% reduction compared to the CS membrane). This was consistent with the ionic conductivity results, which showed that the introduction of CNF(D) filler does not significantly affect the ionic conductivity of the CS membranes (Figure 10d). The increased temperature of 80 °C with humidified oxygen compared to 60 °C with humidified oxygen resulted in an increase in power density for all CS-based and commercial AEMs embedded in the fuel cells, which was expected, since we associated the moderately higher AAEMFC operating temperature with increased P_max_. We found that the fuel cells achieved the maximum P_max_ values with each of the membranes at 80 °C, humidified oxygen, and a fuel mixture of 5 M KOH and 3 M EtOH, as both the increased temperature and higher fuel concentration have a positive effect on fuel cell operation. A significant improvement in power density at 80 °C was observed for the CS membrane with unmodified CNF filler compared to the commercial membrane (62.4 vs. 35.1 mW cm^−2^), which was not detectable for the CS membrane with CNF(D) at these operating conditions. This behavior can be explained by the morphological features observed by the SEM analysis (Figure 8) of the CS-CNF (D) membrane, which showed a strong agglomeration and non-uniform distribution of the CNF(D) filler on the CS base of the membrane, with a poor interweaving of the fibers over the whole surface. At the same conditions of 80 °C and humidified oxygen, as expected, a significant decrease in P_max_ was observed for the less concentrated fuel mixture of 1 M KOH and 1 M EtOH compared to the more concentrated mixture of 3 M KOH and 3 M EtOH for all CS membranes (67–84% lower P_max_) and for the commercial membrane (57% lower P_max_). Based on the membrane tests in a single fuel cell, we found that the most promising CS membrane was the CS membrane with CNF filler.

The fuel cell tests showed that all CS-based AEMs had higher maximum power densities than the commercial Fumatech AEM at 25 °C and 60 °C with humidified oxygen and without oxygen humidification, indicating that all newly prepared CS-based membranes are suitable for DEFC applications at low temperatures. At 80 °C with humidified oxygen and without oxygen humidification, the CS membranes with CNF(D) filler underperformed in comparison to the commercial Fumatech AEM, implying that the CS-based membranes with CNF(D) fillers are not suitable for DEFCs operating at higher temperatures. At the highest temperature, 80 °C, and under both fuel concentrations, the CS-CNF membrane was the best performing membrane, with a P_max_ of 62.4 mW cm^−2^ at 80 °C, humidified oxygen, and a fuel mixture of 5 M KOH and 3 M EtOH, and a P_max_ of 20.6 mW cm^−2^ at 80 °C, humidified oxygen, and a fuel mixture of 1 M KOH and 1 M EtOH. Under all other conditions, the reference CS membrane proved to be the best performing and the CS-CNF membrane the second best. At the higher operating temperature, the CS-CNF membrane achieved the highest maximum power density in this study. The CS and CS-CNF membranes outperformed all the CS-based membranes with CNF(D) fillers. However, the CS-based AEMs with CNF(D) fillers exhibited better mechanical properties, with higher Young’s modulus and tensile strength, except for the CS-CNF-CNF (D)H membrane. The CS-CNF (D) membrane also had significantly lower ethanol permeability than the CS and CS-CNF membranes, which was also comparable to the fuel permeability of the commercial membrane. Although the CS-CNF (D) had the highest IEC among all the CS-based membranes with fillers, it did not produce a higher P_max_ than the CS or CS-CNF membranes when embedded in a fuel cell. For all CS-based membranes with fillers, except CS-CNF (D), we observed an improvement in ionic conductivity compared to the commercial Fumatech AEM, while the reference CS membrane had a lower ionic conductivity than the commercial membrane.

## 4. Conclusions

The trade-off between sustainability, durability, performance, and cost is necessary for the development of new materials that are expected to have far-reaching effects in the long term. In this direction, a series of CS-based composite membranes with (modified) CNF as filler were prepared, and characterized with respect to their potential application in alkaline fuel cells. The CNF was modified efficiently with a DMAOP reagent, which allowed enrichment with quaternary amino groups as hydroxide conductors. The resulting fully bio-based composites were superior to the commercial Fumatech membranes in terms of Young’s modulus, tensile strength, ion exchange capacity, and ionic conductivity, while the CS membrane with modified CNF filler achieved up to the same values in terms of EtOH crossover limitation. In terms of performance in a laboratory fuel cell assembly, an improvement of up to 78% in power density at 80 °C was observed for the CS membrane with neat CNF compared to the commercial Fumatech membrane (62.4 vs. 35.1 mW cm^−2^), as well as a higher maximum power density, even in the experiment at RT. These results demonstrate their potential as sustainable membrane materials for DEFC applications at low temperatures.

## Figures and Tables

**Table 1:** Membrane sample names and corresponding filler content.

Sample	m_CNF_ (g)	m_CNF(D)_ (g)
CS	0	0
CS-CNF	0.375	0
CS-CNF(D)	0	0.125
CS-CNF-CNF(D)	0.375	0.125
CS-CNF-CNF(D)_H_	0.375	0.375

**Table 2:** Temperature areas and percentages of mass loss obtained by TGA analysis for samples of the reference CNF, the hydrolyzed reagent DMAOP at pH = 4 and pH = 8, and the product CNF(D) at pH = 4 and pH = 8.

Sample	T1 (°C)			T2 (°C)			T3 (°C)			∆y1 (%)	∆y2 (%)	∆y3 (%)	∆y (%)
	T1_s_	T1_p_	T1_e_	T2_s_	T2_p_	T2_e_	T3_s_	T3_p_	T3_e_				
CNF	30	80	145	245	350	380	/	/	/	5.2	74.7	/	87.2
DMAOP pH = 4	30	105	175	205	235	265	370	440	525	3.0	14.4	36.6	80.3
DMAOP pH = 8	30	110	175	205	235	265	370	440	525	2.9	14.2	37.0	79.3
CNF(D) pH = 4	30	80	145	255	345	370	/	/	/	4.6	71.2	/	86.2
CNF(D) pH = 8	30	80	180	205	230	245	430	470	510	3.1	10.9	12.0	80.8
				330	375	420					37.2		

**Table 3:** Temperature areas of mass loss and percentage of mass loss for CS membranes with CNF and CNF(D) fillers determined by TGA analysis.

Sample	T1 (°C)			T2 (°C)			T3 (°C)			∆y1 (%)	∆y2 (%)	∆y3 (%)	∆y (%)
	T1_s_	T1_p_	T1_e_	T2_s_	T2_p_	T2_e_	T3_s_	T3_p_	T3_e_				
CS	30	49	110	235	283	345	/	/	/	9.1	35.9	/	85.7
CS-CNF	30	40	105	250	361	400	/	/	/	3.1	53.1	/	72.6
CS-CNF(D)	30	49	135	180	262	340	360	430	520	8.7	36.4	28.8	89.2
CS-CNF-CNF(D)	30	90	120	220	305	367	385	440	500	4.7	50.9	13.0	93.7

## Data Availability

Data available on request from the authors.
